# Association of Exposure to Wildfire Air Pollution With Exacerbations of Atopic Dermatitis and Itch Among Older Adults

**DOI:** 10.1001/jamanetworkopen.2022.38594

**Published:** 2022-10-26

**Authors:** Raj P. Fadadu, Marcus Green, Nicholas P. Jewell, Barbara Grimes, Jason Vargo, Maria L. Wei

**Affiliations:** 1 Department of Dermatology, University of California, San Francisco, San Francisco; 2 Dermatology Service, San Francisco Veterans Affairs Health Care System, San Francisco, California; 3 Department of Medical Statistics, London School of Hygiene & Tropical Medicine, London, United Kingdom; 4 Department of Epidemiology and Biostatistics, University of California, San Francisco, San Francisco; 5 Office of Health Equity, California Department of Public Health, Richmond; 6 Now with Department of Community Development, Federal Reserve Bank of San Francisco, San Francisco, California

## Abstract

This cross-sectional study evaluates the association of exposure to wildfire air pollution with exacerbations of atopic dermatitis and itch among adults aged 65 years or older.

## Introduction

Exposure to smoke from the California Camp Fire in 2018 was associated with a significantly increased number of clinic visits for atopic dermatitis (AD), but not itch, among patients older than 18 years.^1^ Because aging affects the barrier function of skin, including alterations in the acidity and hydration of the stratum corneum, transepidermal water loss, and cytokine-mediated inflammation, aging skin may become less resilient to environmental insults, such as air pollution.^2^ We hypothesized that adults aged 65 years or older would be at greater risk than younger adults for wildfire pollution–related skin exacerbations.

## Methods

As previously described,^1^ we used 3 metrics to characterize air pollution in San Francisco, California: fire status (a binary indicator of whether a wildfire occurred during a week), concentraton of particulate matter with diameters 2.5 μm or smaller (PM_2.5_), and smoke plume density (SPD; range, 0-3, where 0 indicates no smoke and 3 indicates heavy smoke density, as detected on satellite imagery). Data were collected for outpatient dermatology visits for AD or itch at an academic medical center in San Francisco from October 1, 2018, to February 10, 2019 (including the time of the California Camp Fire); from October 1, 2015, to February 10, 2016 (control, no fires); and from October 1, 2016, to February 10, 2017 (control, no fires). Outcome data were aggregated on a weekly basis, segregated by age (18-64 or ≥65 years) and analyzed using generalized Poisson regression. Statistical models included 4 exposure lags and were adjusted for temperature, humidity, year, holiday, and overall patient volume at clinics (eAppendix in the Supplement). Data management and statistical analyses were conducted using Stata, version 16 (StataCorp LLC) and R, version 4.0.5 (R Group for Statistical Computing). The study was approved by the University of California, San Francisco institutional review board, which waived the need for consent because the research was no more than minimal risk to participants; could not practicably be done without the waiver or alteration; could not practicably be done without identifiable information and biospecimens (if applicable); will not adversely affect rights and welfare of participants with the waiver or alteration; and participants will be provided with additional pertinent information after participation, whenever it is appropriate. This cross-sectional study followed the STROBE reporting guideline.

## Results

We assessed 5529 adult visits for AD and 1319 adult visits for itch for 3448 unique patients (2325 women [67.4%]; mean [SD] age, 44.6 [21.1] years) (Table). The California Camp Fire, located 280 km (175 miles) from San Francisco, caused a 9-fold increase in mean weekly PM_2.5_ concentrations in San Francisco during a 2-week period (November 8-21, 2018).^3^

**Table.** Summary Characteristics of the Study Population: Visits and Patients

Characteristic	No. (%)			
	Total^a^	2015-2016 (Control)^b^	2016-2017 (Control)^b^	2018-2019 (Wildfire period)^b^
Adult AD appointments, No.	5529	1477	1790	2262
Aged ≥65 y	1238 (22.4)	399 (27.0)	372 (20.8)	467 (20.6)
Aged 18-64 y	4291 (77.6)	1078 (73.0)	1418 (79.2)	1795 (79.4)
Adult itch appointments, No.	1319	460	422	437
Aged ≥65 y	614 (46.6)	275 (59.8)	163 (38.6)	176 (40.3)
Aged 18-64 y	705 (53.4)	185 (40.2)	259 (61.4)	261 (59.7)
Total adult dermatology clinic appointments, No.	56 575	17 037	18 421	21 117
Unique adult patients, No.	3448	905	1073	1470
Aged ≥65 y	1008 (29.2)	316 (35.0)	299 (27.9)	400 (27.2)
Aged 18-64 y	2440 (70.8)	589 (65.1)	774 (72.1)	1070 (72.8)
Age of all patients, mean (SD), y	44.6 (21.1)	44.8 (21.5)	44.3 (20.3)	44.5 (21.3)
Aged ≥65 y	76.7 (7.7)	76.3 (7.8)	76.8 (7.3)	76.7 (8.0)
Aged 18-64 y	42.4 (12.3)	42.6 (12.7)	42.5 (12.1)	42.3 (12.1)
Female patients	2325 (67.4)	649 (59)	715 (55)	961 (55)
Aged ≥65 y	623 (26.8)	166 (25.6)	195 (27.3)	261 (27.2)
Aged 18-64 y	1702 (73.2)	483 (74.4)	520 (72.7)	700 (72.8)

Abbreviation: AD, atopic dermatitis.

^a^ Percentages represent the fraction of data within the combined data from all 3 time periods.

^b^ Percentages represent the fraction of data within the respective time period (eg, 2015-2016, 2016-2017, or 2018-2019), not the combined data.

The adjusted rate of clinic visits for itch for adults aged 65 years or older during a week with a wildfire was 1.6 (95% CI, 1.1-2.5) times the rates for weeks without a wildfire, for a 0-week lag (Figure). The corresponding rate for adults aged 18 to 64 years was 1.1 (95% CI, 0.8-1.7). A 1-unit increase in mean weekly SPD score was associated with a 1.4 (95% CI, 1.1-1.9) times higher rate of clinic visits for itch for adults aged 65 years or older and no change in the rate of clinic visits for itch for adults aged 18 to 64 years (rate ratio, 1.0 [95% CI, 0.8-1.4]), for a 0-week lag. Similar results were found for the PM_2.5_ exposure metric.

**Figure.**
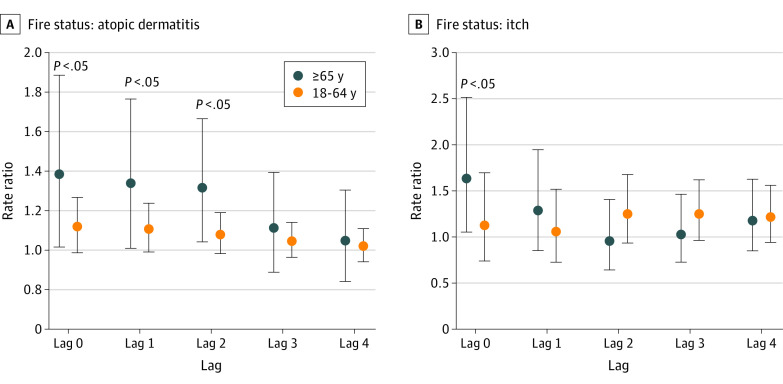
Association of Air Pollution With Clinic Visits for Atopic Dermatitis and Itch for the Fire Status Exposure Metric by Age Group Circles indicate the rate ratio, adjusting for weekly temperature, humidity, year, holiday, and overall patient volume at clinics. Error bars indicate 95% CIs. The lag considers cumulative exposure to air pollution.

The adjusted rate of clinic visits for AD for adults aged 65 years or older during a week with a wildfire was 1.4 (95% CI, 1.1-1.9) times the rate for weeks without a wildfire, for a 0-week lag; the corresponding rate for adults aged 18 to 64 years was 1.1 (95% CI, 1.0-1.3) (Figure). A 1-unit increase in mean weekly SPD score was associated with a 1.3 (95% CI, 1.1-1.6) times higher rate of clinic visits for AD for adults aged 65 years or older and a 1.2 (95% CI, 1.1-1.3) times higher rate for adults aged 18 to 64 years, for a 0-week lag.

## Discussion

We found that during short-term exposure to air pollution from a California wildfire, rates of clinic visits for both AD and itch were significantly increased among adults aged 65 years or older, especially at a 0-week lag (during the wildfire), compared with younger adults. In contrast to the statistically nonsignificant increases in visits for itch for all adults aged 18 years or older in a prior study,^1^ this study demonstrated statistically significantly increased rates specifically for adults aged 65 years or older. This finding suggests that the skin of older adults has a greater vulnerability to air pollution, with rapid outcomes after short-term exposure to air pollution. The increased association of risk for pollution-induced skin exacerbations with older age may be due to age-related molecular processes affecting skin barrier function.^2,4^ Pruritus among older adults can have a multifactorial cause, in which exposure to air pollution may play a role.^5^

This study has some limitations. It was restricted to 1 wildfire, limiting generalizability of results to other wildfires with differing air pollution composition. It also did not include patients with AD and itch at primary care clinics, so observed associations may be underestimated.

Health care systems could increase access to resources, including medical professionals, during wildfires to address AD and itch exacerbations. As wildfires increase in frequency and intensity,^6^ it is important to provide clinical counseling and public health education targeted to older adults on the association of air pollution with skin health.

## Notes

Supplement. **eAppendix.** Data Sources
