# Supplementary material for: Association of Exposure to Wildfire Air Pollution With Exacerbations of Atopic Dermatitis and Itch Among Older Adults
Source: JAMA Netw Open. 2022 Oct 26;5(10):e2238594. doi: 10.1001/jamanetworkopen.2022.38594 (PMC9606843; doi:10.1001/jamanetworkopen.2022.38594)
Supplement: Supplement. — eAppendix. Data Sources [file jamanetwopen-e2238594-s001.pdf]

Supplementary Online Content

Fadadu RP, Green M, Jewell NP, Grimes B, Vargo J, Wei ML. Association of exposure to wildfire air pollution with exacerbations of atopic dermatitis and itch among older adults. *JAMA Netw Open.* 2022;5(10):e2238594. doi:10.1001/jamanetworkopen.2022.38594

eAppendix. Data Sources

This supplementary material has been provided by the authors to give readers additional information about their work.

© 2022 Fadadu RP et al. *JAMA Network Open.*

eAppendix. Data Sources

For $PM_{2.5}$ data, we collected the daily 24-hour average $PM_{2.5}$ concentrations (µg /m^3) from the only Bay Area Air Quality Management District's ground-level air quality monitoring station in San Francisco. Average daily smoke plume density scores were collected from the National Oceanic and Atmospheric Administration (NOAA) Hazard Mapping System (HMS) for Fire and Smoke. Covariate data on the daily mean temperature and relative humidity for San Francisco were obtained from the NOAA Local Climatological Data.

Data on clinic visits were collected using codes from the *International Statistical Classification of Diseases and Related Health Problems, Tenth Revision* (*ICD-10*). The *ICD-10* codes used for eczema/AD were L30.9, L20.82, L20.84, L20.9, H01.139, H01.136 L, H01.133 R, L28.0 and for itch were L29.9 and L29.8. Information on patient age and sex was then extracted from the electronic medical record system.

Statistical Analysis:

We calculated the weekly sum of clinic visits (adults age 18-64 and ≥65) for AD and itch and the weekly average $PM_{2.5}$ concentration, smoke plume density score, temperature, relative humidity, and age of patients. We used a generalized Poisson regression model:

$$f(y_i, \mu_i, \alpha) = \left(\frac{\mu_i}{1 + \alpha\mu_i}\right) \frac{(1 + \alpha y_i)^{y_i - 1}}{y_i!} \cdot e^{\left[\frac{-\mu(1+\alpha y_i)}{1+\alpha\mu_i}\right]},$$

where $Y_i, (i = 1, 2, 3, \dots)$ is the number of appointments for AD or itch and α is a parameter which accounts for the variation in the number of appointments. The probability function of Y_i is given by: $y_i = 0, 1, 2, \ldots$, and $\mu_i = \mu_i(x_i) = exp(x_i\beta)$, where x_i is a $(k - 1)$ dimensional vector of covariates including air pollution metric, average temperature, average humidity, and holiday week. β is a k-dimensional vector of regression parameters.

© 2022 Fadadu RP et al. *JAMA Network Open.*

Each outcome was analyzed in models that included a specific exposure metric as the primary predictor: fire, plume density score, or $PM_{2.5}$. Four 1-week cumulative exposure lags were constructed and analyzed independently in separate models. Covariates in the models included average temperature, relative humidity, patient age, and time period (2015-2016, 2016-2017, or 2018-2019). In addition, each model included an offset variable, the logarithm of the weekly total number of dermatology clinic visits for any skin health-related concern. Lastly, the models included a holiday week indicator variable. Data management and statistical analyses were conducted using STATA 16 (StataCorp LLC., College Station, Texas, U.S.) and R (Foundation for Statistical Computing, Vienna, Austria). The reporting of the methods and results of this study follow the Strengthening the Reporting of Observational Studies in Epidemiology (STROBE) guidelines for observational cross-sectional studies. The study was approved by the UCSF Institutional Review Board.

© 2022 Fadadu RP et al. *JAMA Network Open*.
